# Correction: Randomized controlled trial of the effectiveness of olive and black seed oil combination on pain intensity and episiotomy wound healing in primiparous women: A study protocol

**DOI:** 10.1371/journal.pone.0308811

**Published:** 2024-08-08

**Authors:** The PLOS ONE Staff

There is an error in affiliation 1 for authors Romina Fili and Sana Nazmi. The correct affiliation 1 is: Student Research Committee, Babol University of Medical Sciences, Babol, Islamic Republic of Iran. The publisher apologizes for this error.

There is an error in the Acknowledgments section. The correct Acknowledgments is: The authors would like to thank the Student Research Committee and Deputy of Research and Technology of Babol University of Medical Sciences and the mothers who will participate in this study (Study code: 724134467).
