# Supplementary material for: Randomized controlled trial of the effectiveness of olive and black seed oil combination on pain intensity and episiotomy wound healing in primiparous women: A study protocol
Source: PLoS One. 2024 May 15;19(5):e0302161. doi: 10.1371/journal.pone.0302161 (PMC11095949; doi:10.1371/journal.pone.0302161)
Supplement: S1 File — (PDF) [file pone.0302161.s002.pdf]

Babol University of Medical Sciences

Proposal title: The effectiveness of olive and black seed oil combination in pain intensity and episiotomy wound healing in primiparous women referred to hospitals under the auspices of Babol University of Medical Sciences

Introduction

Episiotomy is an incision in the area between the perineum and the anus, performed to enlarge the perineum and facilitate the process of natural childbirth, especially in primiparous women (1-3). This intervention is associated with side effects such as pain (3-5). Studies have shown that approximately 30% of women experience pain in the first two weeks, and 7% experience it up to 3 months after natural delivery (6). Due to the lack of direct observation of the mother and the proximity of the wound area to the anus, there is a possibility of wound infection (7-9). Discomfort after episiotomy affects the quality of life of both the mother and the baby (3, 10, 11). Considering that the baby requires the mother's presence and support for growth and development, especially in the early days after birth, it is crucial to pay attention and provide support to mothers who have undergone an episiotomy (7, 12). Since Iran's current policy is to increase the birth rate and population, more attention should be paid to prenatal and post-pregnancy care, especially for vaginal childbirth. An unpleasant experience during childbirth can affect a mother's future pregnancies and may lead to her deciding not to have more children, ultimately reducing population growth (6). On the other hand, if episiotomy is not used, there is a risk of perineal tears that may cause problems for the mother, such as a reduced quality of sexual life (4, 13) and difficulties with breastfeeding (2, 14). To alleviate the pain intensity of episiotomy and facilitate its faster healing, various treatment methods, including oral, rectal, and sitz baths, are used (12, 15). Due to the high costs and complications of treatment with chemical drugs (4), it is better to use herbal drugs, as recommended by the World Health Organization (16). It appears that plant oils, such as olive oil and black seed oil, may be effective in promoting the healing and repair of perineal wounds (16, 17). According to studies, many medicinal plants, especially olive oil, can accelerate wound healing and reduce pain and complications caused by episiotomy (14, 18). Olive oil is rich in substances that provide it with antimicrobial, antifungal, and anti-inflammatory properties (19, 20). These include phenolic compounds that promote cell repair, antioxidants that help reduce inflammation, and oleocanthal that can alleviate pain (12, 18, 21). Olive oil has been found effective in treating skin diseases, suppressing the growth of viruses and bacteria, and promoting wound healing (12, 22).

Black seed contains 30% to 40% oil and is particularly rich in a type of antioxidant called thymoquinone (23, 24), as well as phenols, linoleic acid, and oleic acid, which have been the subject of various studies demonstrating their positive effects on wound healing (25). Studies have shown that black seed is suitable for promoting wound healing in the human body and can help prevent infections (26, 27). Furthermore, adding black seed oil to olive oil enhances its antioxidant properties (28). Given the limited number of human clinical studies on black seed oil and the scarcity of research regarding the local effectiveness of the combination of olive oil and black seed

oil in alleviating pain and promoting episiotomy wound healing, the present study aims to evaluate the effectiveness of the combination of olive and black seed oil in reducing pain intensity and facilitating episiotomy wound healing in primiparous women.

Review of texts

1. Reda M. Hables (2021) conducted a study with the aim of comparing and investigating the effect of olive oil, lavender and placebo on 120 women at Al-Shatabi Hospital in Egypt. The samples were randomized into three groups receiving lavender, olive oil or vegetable oil as a placebo. The first group was treated with 10 drops of two percent lavender oil in five liters of water, the second group was treated with olive oil sitting bath (10 drops in five liters of water) and the third group was treated with a placebo with the same method as the second group for 14 days. Pain intensity was assessed by Visual Analog Pain Scale (VAS) and redness, edema and discharge were assessed by REEDA[1] scale. On the first day, the pain score and REEDA score were not significantly different between the three groups, but there was a significant difference between the three groups on the fifth, ninth and fourteenth days after episiotomy. The result of the study showed that the use of olive or lavender oil is effective in reducing the damage to the perineum caused by episiotomy and its repair (6). This study was in the form of a sitting bath in warm water, but the present study will be in the form of topical application of olive oil and black seed.
2. Nahid Barati and Masoumeh Kordi (2020) conducted a clinical trial study titled the effect of black seed cream on episiotomy pain. This study was conducted on 124 primiparous women of Vali Asr Hospital in Birjand. The samples were randomly divided into three groups: black seed cream, placebo (cold cream) or routine care. Four hours after episiotomy, both placebo and black seed cream groups used the creams in the amount of 20 grams twice a day for 10 days. And the control group received usual care. Pain intensity was assessed before the intervention, 12 hours after the intervention, and on the seventh and tenth days after the intervention by the McGill questionnaire. There was no significant difference in the pain intensity in the three groups before the intervention and 12 hours after, but on the seventh and tenth days after the intervention, the pain intensity in the group receiving black seed cream was far less than the other two groups. The results of this study showed that black seed cream reduces the intensity of pain caused by episiotomy. Therefore, it can be used as a non-pharmacological method (16). This study examined the effectiveness of black seed cream only on pain intensity. However, the present study will investigate the effectiveness of the combination of black seed oil with olive oil on pain intensity and episiotomy wound healing.
3. M. Kaviani et al. (2017) conducted a study with the aim of determining the effect of olive leaf extract ointment on reducing the intensity of episiotomy pain and its recovery. This

[1] Redness, Edema, Ecchymosis, Drainage, Approximation

randomized trial was conducted on 90 women including three groups of 30 people who used olive leaf ointment, placebo and betadine solution three times a day for 10 days locally in the episiotomy area. Data were collected 4 hours after delivery and on the third, seventh, tenth and fourteenth days after delivery. Wound healing was evaluated by REEDA scale and pain intensity was evaluated by Visual Analog Pain Scale. The findings of the study showed that there was a significant difference between the olive leaf extract ointment group and other groups in terms of wound healing and pain. And this ointment is effective in reducing pain intensity and early complications of episiotomy (14).

4. A study by Amani R. et al. (2015) was conducted on 90 primiparous women in hospitals affiliated to Gilan University of Medical Sciences, Iran. The aim of this study was to compare the effect of cold gel pad and topical olive oil on episiotomy wound healing. The samples were randomly placed in two groups receiving cold gel pads for 20 minutes at 12 hours after delivery and if needed for 10 days or topical olive oil twice a day at 12 hours and 10 days after delivery. Episiotomy wound healing was evaluated at 12 hours after delivery and on the fifth and tenth days with the REEDA scale. 10 days after delivery, there was a significant difference between the two groups in terms of redness, and based on this study, olive oil can accelerate episiotomy wound healing (17).
5. Bahmanesh et al. (2013) conducted a clinical trial with the aim of investigating the effect of olive oil sitz bath on improving perineal injury after childbirth. This study was conducted on 60 women who had mediolateral episiotomy or first- or second-degree tears. The samples were randomly divided into two treatment groups with olive oil sitz bath or distilled water sitz bath control group. The intervention group used olive oil sitting bath for 10 minutes twice a day for 10 days, 24 hours after delivery. In this way, five drops of olive oil were poured into five liters of water. The control group also used a sitting bath of distilled water in the same way. The data were evaluated in the first two hours after delivery, on the fifth and tenth days, using the visual pain scale and REEDA. The findings of the study showed that there was no significant difference in pain intensity and wound healing between the studied groups in the first two hours. However, on the fifth and tenth days, there was a significant difference in terms of pain intensity and wound healing between the two groups of olive oil and distilled water. As a result, it was suggested to use olive oil sitting bath in warm water for episiotomy care (12).

Objective and assumptions

General goal

Determining the effectiveness of olive and black seed oil combination in pain intensity and episiotomy wound healing in prim parous women referred to hospitals under the auspices of Babol University of Medical Sciences

Specific objectives

• Comparison of the change in the average intensity of episiotomy pain in three groups receiving the combination of olive oil and black seed, olive oil alone and the routine care group in the first 10 days after delivery
• Comparison of the average change of episiotomy wound healing in three groups receiving the combination of olive oil and black seed and the routine care group in the first 10 days after delivery
• Comparison of side effects caused by drug use (burning and itching, episiotomy wound opening and need for re-repair) in three groups

Practical objectives

If the combination of olive oil and black seed oil or olive oil alone is effective with a local method to reduce pain intensity and heal the episiotomy wound after natural childbirth, these substances can be used so that mothers can have a pleasant experience of childbirth with faster healing of the episiotomy wound have a natural the pleasant experience of natural childbirth will lead to the promotion of this importance and increase the motivation for having children again, which is part of the country's demographic policies.

Assumptions

• The average change of episiotomy pain intensity is different in the three groups receiving the combination of olive oil and black seed, olive oil alone, and the routine care group for the first 10 days after delivery.
• Comparison of the average change of episiotomy wound healing in the three groups receiving the combination of olive oil and black seed and the routine care group is different in the first 10 days after delivery.
• Comparison of side effects caused by drug use (burning and itching, episiotomy wound opening and the need for re-repair) are different in the three groups.

Table of variables

Variable name	Measurement method	variable role	variable scale	Variable type	Functional definition
Drug intervention	questionnaire	Independent	nominal	Qualitative	done/not done
Severity of episiotomy pain	10cm pain ruler	Dependent	relative	Quantitative/Continuous	Based on the person's statements, zero means the least pain and 10 means the most possible pain
Episiotomy wound healing	REEDA scale	Dependent	relative	Quantitative/Continuous	Based on measuring the variables of swelling, bruising, redness and the distance between the two edges of the episiotomy wound in the lithotomy position. The score of each part is 0 to 3 and the total score is from 0 to 15
Mrs. age	year	contextual variable	relative	Quantitative/Continuous	Counting the years of life based on the date of birth according to the person's statement
Mrs. Education	Illiterate, elementary, middle school, high school, university	contextual variable	Rank variable	Qualitative	Based on the last educational qualification and according to the person's statements
Address	City, village	contextual variable	nominal	Qualitative	Based on individual statements
husband's age	year	contextual variable	Rank variable	Quantitative/Continuous	Counting the years of life based on the date of birth according to the person's statement
Woman's job	Working at home, working outside the home, unemployed	contextual variable	nominal	Qualitative	Based on individual statements

Husband’s job	Unemployed, farmer, employee, freelancer, specialist (engineering, medicine, judgment, etc.), manager, student, others	contextual variable	nominal	Qualitative	Based on individual statements
The economic situation	Easy, not easy, not hard, sometimes hard, hard, I don't know	contextual variable	Rank variable	Qualitative	Based on individual statements
height		contextual variable	relative	Quantitative/Continuous	Based on individual statements
Weight		contextual variable	relative	Quantitative/Continuous	Based on individual statements
Having a fever during labor	no, yes	intervening variable	nominal	Qualitative	Based on individual statements
Receive antibiotics in labor	no, yes	intervening variable	nominal	Qualitative	Based on individual statements
Hospitalization of the baby in Special care unit	no, yes	contextual variable	nominal	Qualitative	Based on individual statements
Receiving antibiotics in the postpartum period	no, yes	intervening variable	nominal	Qualitative	Based on individual statements
painkiller	Number of painkillers used	intervening variable	nominal	Qualitative	Based on individual statements

method

Study population and sampling method

This randomized clinical trial will be conducted as a parallel-group, non-inferiority study on postpartum women who have undergone episiotomy in the hospitals of Babol city. The samples

will be included in the study by easy sampling method and then they will be randomly assigned into three groups.

Sample size

The sample volume was calculated at the confidence level of 95%, the power of the test was 80%, and according to the information of the article by Kurdi et al. (16) with values of σ1=1.5, μ1=5, σ2=2.2, and μ2=6.4, with the following formula and assuming dropout During the implementation, the number of 36 samples in each group is estimated.

$$n = \frac{(Z_{1-\alpha/2} + Z_{1-\beta})^2 (S_1^2 + S_2^2)}{(\mu_1 - \mu_2)^2}$$

Statistical analysis

The analysis method used in this study is intention-to-treat analysis, ensuring the maintenance of the random allocation process. After collecting the data in the first stage, the basic variables of the patients in the intervention and control groups will be compared to ensure the desirability of the random allocation process. SPSS version 20 software and ANOVA, chi-square, repeated measurements and multilevel mixed-effects linear regression will be used to analyze the primary results. For comparing pain intensity and episiotomy wound healing after the intervention in the 3 groups, the analysis of covariance (ANCOVA) test will be used, taking into account the measurements taken prior to the intervention. Multilevel linear regression will be conducted to adjust for confounding variables, including demographic, midwifery, and delivery-related factors. Additionally, the effect size will be demonstrated through mean differences and 95% CIs. A significance level of P < 0.05 will be considered as the threshold for statistical significance

Data Collection and Management

This research will be conducted after obtaining permission from the Ethics Committee of Babol University of Medical Sciences, registering with the Iranian Registry of Clinical Trials, and obtaining the necessary sampling permits. After contacting the midwifery department of the hospitals affiliated with Babol University of Medical Sciences, the researcher invites eligible women to participate in the study. The researcher will first explain the study's purpose and the nature of the intervention to the women. The researcher ensures the confidentiality of their information, clarifies her responsibility for any treatment complications, and assures them that the treatment interventions will not change if they choose not to participate. Then, by obtaining the informed consent of the qualified, she will conduct convenience sampling.

Entry criteria for this study: primiparous women, natural vaginal delivery with episiotomy, literacy, and BMI < 30.

Exclusion criteria from this study: unwillingness of participants to continue participating in the study, fourth-degree tear, history of dermatitis, history of allergy and eczema diagnosed related to plants, and history of gestational diabetes and overt diabetes.

The researcher will select and randomly assign the samples to two intervention groups and one routine care group according to the study entry criteria. Eligible people are placed in one of three groups A: 36 people, B: 36 people, and C: 36 people, using permuted block randomization and open label. 18 blocks of 6 will be produced to create a sequence of size 108. In order to keep the random allocation list secret, a special code will be assigned to each of the intervention groups, which only the main implementer of the project will be aware of. Also, the second moderator, who is a student and study evaluator, and the person who performs the statistical analysis, will not know about the codes assigned to the drugs. The intervention group consisted of two groups of mothers, one group will receive black seed and olive oil and the other group will receive olive oil alone. To prepare the product, cold-pressed black seed oil is used, as well as standard extra-virgin olive oil (according to the national standard). Standard extra virgin olive oil is prepared by a company with pharmaceutical grade (Pisgaman Chemi). Cold-pressed black seed oil is obtained from pharmaceutical company (Barij Essential Oil). Both oils are mixed in equal proportions and packaged (29). Both products have approved specifications in terms of microbial and pollution control standards, chemical and physical control. The process of mixing and packaging of two oils is done under hygienic conditions in the traditional pharmacy laboratory of the Faculty of Traditional Medicine. All study subjects will be educated on following post-delivery health tips and caring for episiotomy wounds. The intervention groups (one group receiving olive oil plus black seed oil and the other receiving olive oil alone) will also receive instructions on how to use the containers containing the oils. Women in the intervention groups will use the oils 24 hours after delivery, applying 10 drops topically 3 times a day for 10 days (12). The participants will be instructed to wash and dry their perineum before applying the oils. They should then wear gloves and slowly apply 10 drops of oil to the perineum, massaging it in with their hands. The control group will receive only routine care. The researcher also examines the severity of perineal pain and episiotomy wound healing in both groups, the first 24 hours after delivery and ten days after that.

Given that each milliliter contains 15 drops, and mothers are required to use the oils 3 times a day, approximately 30 mL of each type of oil will be provided to mothers for the duration of 10 days.

To measure the intensity of episiotomy pain, a 10-cm pain ruler scale (6, 12) will be used, the zero mark of which means no pain and the mark of 10 is the worst possible pain. Episiotomy wound healing will be assessed by the researcher using the Rida scale (6, 12, 17). In this way, the participant will be placed in a lithotomy position and the variables of redness, edema, ecchymosis, discharge and approximation between the two edges of the episiotomy wound will be scored based on the REEDA scale (12).

Secondary outcomes include the monitoring of symptoms such as burning and itching, as well as the occurrence of episiotomy opening and the need for painkillers. These secondary outcomes will be assessed through a combination of examinations conducted by the researcher and self-reports provided by the mothers. Additionally, any side effects reported by each participant will be carefully reviewed, and the total number of side effects within each group will be documented.

Primary outcome

The assessment of pain intensity in the episiotomy area and the evaluation of episiotomy wound healing

Secondary outcomes

Burning and itching, as well as the occurrence of episiotomy opening and the need for painkillers

In this research, 3 questionnaires will be administered within the first 24 hours and 10 days after delivery.

Data collection tools

The data collection tool will be Demographic Characteristics Questionnaire, REEDA scale and Visual Analog Scale.

Demographic Characteristics Questionnaire: This questionnaire comprises 22 questions that gather information such as age, education level, place of residence, employment status, height, weight, number of pregnancies and abortions, duration of hospitalization during labor, receipt of antibiotics during labor and post-delivery, and hospitalization of the baby in the intensive care unit. The questionnaire was designed based on the research objectives to identify potential confounding variables.

Visual Analog Scale for Measuring Episiotomy Pain Intensity: The Visual Analog Scale, also known as the McGill Pain Ruler, is a linear scale used for pain assessment. It consists of a 10-cm ruler, with 0 indicating no pain and 10 indicating the most severe pain possible, and between these 2 endpoints, the scale is divided into 3 levels. Perineal pain intensity refers to the pain experienced by mothers in their perineal area and is self-reported before and after the intervention.

The Redness, Edema, Ecchymosis, Discharge, and Approximation Scale: The REEDA (Redness, Edema, Ecchymosis, Discharge, and Approximation) Scale is a standardized tool for assessing episiotomy wound healing, including variables such as swelling, bruising, discharge, redness, and the distance between the two edges of the episiotomy wound. Each of these variables is scored on a scale from 0 to 3. The researcher records the scores for each variable separately after observing and examining the patient both before and after the examination. The overall score on the scale ranges from 0 to 15, with higher scores indicating less favorable wound healing and lower scores indicating better wound healing.

Secondary outcomes assessment checklist: Secondary outcomes will be determined by a checklist that will be completed by mothers on a daily basis during the first ten days after delivery and will be examined by the researcher.

Possible implementation problems

Not using sterile tape and using eye examination.

Some mothers may not come for examination on the 10th day. (For example, due to hospitalization of the baby)

Forecasting the timing of project implementation stages

Stage length in months	Brief description of the selection process
2 months	Setting up the plan
16 months	Implementation steps of the plan
3 months	Data analysis
3 months	Final project report

Ethical considerations

• Necessary permission will be obtained from the research ethics committee of the honorable research vice president of the university in order not to contradict the study with research ethics.

• By obtaining a written letter of introduction from Babol University of Medical Sciences, the researcher will introduce himself to the relevant officials of the hospitals covered by the university.

• The researcher will clearly present his goals to the officials of the medical centers and the subjects of the research, verbally and in writing, and will get permission to conduct the research.

• Written consent will be obtained from the people participating in the research.

• Subjects will be free to agree or disagree to participate in the study or withdraw from it.

•All people participating in the research will be assured of the confidentiality of their information, and they can leave the study for any reason, such as choosing other methods of episiotomy wound care during the research.

References

.١ Tara F, Golmakani N, Motlagh ER, Assili J, Shakeri M. P284 The effects of turmeric (Curcuma longa L) ointment on healing of episiotomy site in primiparous women. International Journal of Gynecology & Obstetrics. 2009;107:S493-S4.

.٢ Vakilian K, Atarha M, Bekhradi R, Chaman R. Healing advantages of lavender essential oil during episiotomy recovery: a clinical trial. Complementary therapies in clinical practice. 2011;17(1):50-3.

.٣ Moradi M, Niazi A, Mazloomi E, Mousavi SF, Lopez V. Effect of lavender on episiotomy wound healing and pain relief: a systematic review. Evidence Based Care Journal. 2020;10(1):61-9.

.٤ Sabzaligol M, Safari N, Baghcjeghi N, Latifi M, Bekhradi R, Taghizadeh M, et al. The effect of Aloevera gel on prineal pain & wound healing after episiotomy. Complementary Medicine Journal. 2014;4(2):766-75.

.٥ Shayan A, Sourinezhad H, Barzegar F, Ahmadinia H, Masoumi Z, Moradkhani S, et al. The effect of olive oil and honey combination on episiotomy wound healing and pain relief: A randomized clinical trial. Current Women's Health Reviews. 2020;16(2):145-51.

.٦ Hables RM. Effect of Olive Oil, Lavender Oil and Placebo on Pain Intensity and Healing of Episiotomy in Women. Tanta Scientific Nursing Journal. 2021;20(1):47-63.

.٧ Mardani F, Hadizadeh-Talasaz F, Bahri N. Effect of medicinal plants on episiotomy wound healing in Iran: a systematic review study. The Iranian Journal of Obstetrics, Gynecology and Infertility. 2020;23(5):79-94.

.٨ Nikpour M, Delavar MA, Khafri S, Ghanbarpour A, Moghadamnia AA, Esmaeilzadeh S, et al. The use of honey and curcumin for episiotomy pain relief and wound healing: A three-group double-blind randomized clinical trial. Nursing and Midwifery Studies. 2019;8(2):64.
.٩ Torkashvand S, Jafarzadeh-Kenarsari F, Donyaei-Mobarrez Y, Chaboki BG. Effectiveness of Olea Herbal Ointment on Episiotomy Wound Healing Among Primiparous Women: A Randomized Clinical Trial. Jundishapur Journal of Natural Pharmaceutical Products. 2021;16.(٣)
.١٠ Sheikhan F, Jahdi F, Khoei EM, Shamsalizadeh N, Sheikhan M, Haghani H. Episiotomy pain relief: Use of Lavender oil essence in primiparous Iranian women. Complementary therapies in clinical practice. 2012;18(1):66-70.
.١١ Golozar S, Namjouian F, Latifi SM, Mirahi A. Evaluating the effect of oral bromelain (pineapple) on episiotomy wound healing in primiparus women. KAUMS Journal (FEYZ). 2011;15(2):84-90.
.١٢ Behmanesh F, Aghamohammadi A, Zeinalzadeh M, Khafri S. Effects of olive oil sitz bath on improvement of perineal injury after delivery. Koomesh. 2013;14.(٣)
.١٣ Hartmann K, Viswanathan M, Palmieri R, Gartlehner G, Thorp J, Lohr KN. Outcomes of routine episiotomy: a systematic review. Jama. 2005;293(17):2141-8.
.١٤ Kaviani M, Sepasi S, Azima S, Emamghoreishi M, Asadi N, Haghpanah S. The effects of olive leaf extract ointment on pain intensity and early maternal complications in primiparous women. Int J Pharm Pharm Sci. 2017;9(7):31-4.
.١٥ Mohamed H, El-Nagger NS. Effect of self perineal care instructions on episiotomy pain and wound healing of postpartum women. J Am Sci. 2012;8(6):640-50.
.١٦ Barati N, Kordi M. The effect of black seed cream on episiotomy pain in primiparous women: a clinical trial study. The third international conference on new findings in obstetrics, gynecology, childbirth and infertility2020.
.١٧ Amani R, Kariman N, Mojab F, Alavi H, Majidi S. Comparison of the effects of cold compress with gel packs and topical olive oil on episiotomy wound healing. Journal of Babol University of Medical Sciences. 2015;17(11):7-12.
.١٨ Abooei Mehrizi M, Eidi A, Mortazavi P. Study of effect of olive oil on re-epithelialization of epithelial tissue in excision wound healing model in rats. Journal of Comparative Pathobiology. 2016;13(2):1875-84.
.١٩ Badiu D, Rajendram R. Chapter 33 - Effect of olive oil on the skin. In: Preedy VR, Watson RR, editors. Olives and Olive Oil in Health and Disease Prevention (Second Edition). San Diego: Academic Press; 2021. p. 401-13.
.٢٠ Donato-Trancoso A, Monte-Alto-Costa A, Romana-Souza B. Olive oil-induced reduction of oxidative damage and inflammation promotes wound healing of pressure ulcers in mice. Journal of dermatological science. 2016;83(1):60-9.
.٢١ Raisi A, Farjanikish G, Salahi P. Comparing Healing Effects of olive leaf extract ointment and dermaheal ointment on cutaneous wound in diabetic rats. Journal of Mazandaran University of Medical Sciences. 2019;29(173):11-21.
.٢٢ Bayir Y, Un H, Ugan RA, Akpinar E, Cadirci E, Calik I, et al. The effects of Beeswax, Olive oil and Butter impregnated bandage on burn wound healing. Burns. 2019;45(6):1410-7.
.٢٣ Randhawa MA, Alghamdi MS. Anticancer activity of Nigella sativa (black seed)—a review. The American journal of Chinese medicine. 2011;39(06):1075-91.
.٢٤ Vedaei S, Aarabi A. An investigation on the effect of cold press and soxhlet methods of extracting Iranian black seed oil on physicochemical and antioxidant properties. Journal of Food Processing and Preservation. 2021;13(1):89-101.
.٢٥ Sallehuddin N, Nordin A, Bt Hj Idrus R, Fauzi MB. Nigella sativa and its active compound, thymoquinone, accelerate wound healing in an in vivo animal model: A comprehensive review. International journal of environmental research and public health. 2020;17(11):4160.
.٢٦ Zaid MHM, Hanafi MF, Haris MS. A review of black seed extract as an agent in the wound healing process. Journal of Pharmacy. 2021;1(2):87-96.

.۲۷ Gumus ZP, Guler E, Demir B, Barlas FB, Yavuz M, Colpankan D, et al. Herbal infusions of black seed and wheat germ oil: Their chemical profiles, in vitro bio-investigations and effective formulations as phyto-nanoemulsions. Colloids and Surfaces B: Biointerfaces. 2015;133:73-80.
.۲۸ Shaheen M, Dolganova N, Shinkar EV, Sukhenko LT, Astafieva OV. STUDY OF BIOTECHNOLOGY RAISE ANTIOXIDANT PROPERTIES OF OLIVE OIL AND BLACK SEED OIL. Carpathian Journal of Food Science & Technology. 2020;12.(۲)
.۲۹ Yaman I, Durmus A, Ceribasi S, Yaman M. Effects of Nigella sativa and silver sulfadiazine on burn wound healing in rats. Veterinarni Medicina. 2010;55(12):619-24.
