# Supplementary material for: Randomized controlled trial of the effectiveness of olive and black seed oil combination on pain intensity and episiotomy wound healing in primiparous women: A study protocol
Source: PLoS One. 2024 May 15;19(5):e0302161. doi: 10.1371/journal.pone.0302161 (PMC11095949; doi:10.1371/journal.pone.0302161)
Supplement: Attachment — Submitted filename: PONE-D-23-34640 et 22Nov 2023 .pdf [file pone.0302161.s003.pdf]

PLOS ONE

Randomized controlled trial of the effectiveness of olive and black seed oil combination in pain intensity and episiotomy wound healing in primiparous women: study protocol

--Manuscript Draft--

**Manuscript Number:**	PONE-D-23-34640
**Article Type:**	Study Protocol
**Full Title:**	Randomized controlled trial of the effectiveness of olive and black seed oil combination in pain intensity and episiotomy wound healing in primiparous women: study protocol
**Short Title:**	olive and black seed oil combination in pain intensity and episiotomy wound healing in primiparous women:
**Corresponding Author:**	Fereshteh Behmanesh<br>Babol University of Medical Science<br>Babol, Mazandaran IRAN, ISLAMIC REPUBLIC OF
**Keywords:**	Olive oil; Black seed oil; Episiotomy; Wound healing; Clinical protocol
**Abstract:**	Background<br><br>Episiotomy is associated with side effects, such as pain and wound infection. Also, Discomfort after episiotomy affects the quality of life of mother and baby. Medicinal herbs are one alternative methods for the treatment of episiotomy wound. This study will investigate the effectiveness of olive and black seed oil combination in pain intensity and episiotomy wound healing in primiparous women.<br><br>Methods<br><br>This randomized clinical trial will be done with two interventions and one control groups on primiparous women have normal delivery with episiotomy. women in the intervention group (One group, olive oil plus black seed oil and another group olive oil alone) will use a glass containing oils with a dose of 10 drops locally and 3 times a day 24 hours after delivery, for 10 days. The control group will receive only routine care. Data will be collected through demographic characteristic questionnaire, REEDA scale and visual analog scale. In order to determine and compare the effect of pharmacological interventions on the severity of pain and episiotomy wound healing in groups, an ANOVA analysis of variance test with repeated measures will be used by SPSS software version 22.<br><br>Discussion<br><br>The results of this study will show the effects of olive and black seed oil combination and olive oil alone in pain intensity and episiotomy wound healing in primiparous women. The beneficial effects likely to be found in the trial of these oils may be beneficial for women who have undergone episiotomy.
**Order of Authors:**	Romina fili
	Fereshteh Behmanesh
	Maryam Nikpour
	Zahra Memariani
	Sana Nazmi
**Opposed Reviewers:**	Mouloud Aghajani Delavar<br>Babol University of Medical Science<br><br>She has conflict of interest with us.
**Additional Information:**	
**Question**	**Response**

Powered by Editorial Manager® and ProduXion Manager® from Aries Systems Corporation

**Financial Disclosure**<br><br>Enter a financial disclosure statement that describes the sources of funding for the work included in this submission. Review the submission guidelines for detailed requirements. View published research articles from *PLOS ONE* for specific examples.<br><br>This statement is required for submission and **will appear in the published article** if the submission is accepted. Please make sure it is accurate.<br><br>**Funded studies**<br>Enter a statement with the following details:<br>• Initials of the authors who received each award<br>• Grant numbers awarded to each author<br>• The full name of each funder<br>• URL of each funder website<br>• Did the sponsors or funders play any role in the study design, data collection and analysis, decision to publish, or preparation of the manuscript?<br><br>Did you receive funding for this work?	Yes
Please add funding details.<br>as follow-up to "**Financial Disclosure**<br><br>Enter a financial disclosure statement that describes the sources of funding for the work included in this submission. Review the submission guidelines for detailed requirements. View published research articles from *PLOS ONE* for specific examples.<br><br>This statement is required for submission and **will appear in the published article** if the submission is accepted. Please make sure it is accurate.	This study was financially supported by Babol University of Medical Sciences. Award Number: 724134467. F.B will receive the fund.<br>URL: https://www.mubabol.ac.ir/<br>This funder contributed to the approval of the study.

Powered by Editorial Manager® and ProduXion Manager® from Aries Systems Corporation

**Funded studies**<br>Enter a statement with the following details:<br>• Initials of the authors who received each award<br>• Grant numbers awarded to each author<br>• The full name of each funder<br>• URL of each funder website<br>• Did the sponsors or funders play any role in the study design, data collection and analysis, decision to publish, or preparation of the manuscript?<br><br>Did you receive funding for this work?"	
Please select the country of your main research funder (please select carefully as in some cases this is used in fee calculation).<br>as follow-up to "**Financial Disclosure**<br><br>Enter a financial disclosure statement that describes the sources of funding for the work included in this submission. Review the submission guidelines for detailed requirements. View published research articles from *PLOS ONE* for specific examples.<br><br>This statement is required for submission and **will appear in the published article** if the submission is accepted. Please make sure it is accurate.<br><br>**Funded studies**<br>Enter a statement with the following details:<br>• Initials of the authors who received each award<br>• Grant numbers awarded to each author<br>• The full name of each funder<br>• URL of each funder website<br>• Did the sponsors or funders play any role in the study design, data collection and analysis, decision to publish, or preparation of the manuscript?<br><br>Did you receive funding for this work?"	IRAN, ISLAMIC REPUBLIC OF - IR
**Competing Interests**	The authors have declared that no competing interests exist.

Powered by Editorial Manager® and ProduXion Manager® from Aries Systems Corporation

Use the instructions below to enter a competing interest statement for this submission. On behalf of all authors, disclose any competing interests that could be perceived to bias this work—acknowledging all financial support and any other relevant financial or non-financial competing interests.<br><br>This statement is required for submission and **will appear in the published article** if the submission is accepted. Please make sure it is accurate and that any funding sources listed in your Funding Information later in the submission form are also declared in your Financial Disclosure statement.<br><br>View published research articles from *PLOS ONE* for specific examples.<br><br>**NO authors have competing interests**<br><br>Enter: *The authors have declared that no competing interests exist.*<br><br>**Authors with competing interests**<br><br>Enter competing interest details beginning with this statement:<br><br>*I have read the journal's policy and the authors of this manuscript have the following competing interests: [insert competing interests here]*<br><br>* typeset	
**Ethics Statement**<br><br>Enter an ethics statement for this submission. This statement is required if the study involved:<br><br>• Human participants<br>• Human specimens or tissue<br>• Vertebrate animals or cephalopods<br>• Vertebrate embryos or tissues	The study is approved by the Ethics Committee of Babol University of Medical Sciences (IR.MUBABOL.REC.1401.145). All methods will perform in accordance with the relevant guidelines and regulations (Declaration of Helsinki) and Informed consent obtained from participants.

• Field research<br><br>Write "N/A" if the submission does not require an ethics statement.<br><br>General guidance is provided below. Consult the submission guidelines for detailed instructions. **Make sure that all information entered here is included in the Methods section of the manuscript.**	

**Format for specific study types**<br><br>**Human Subject Research (involving human participants and/or tissue)**<br>• Give the name of the institutional review board or ethics committee that approved the study<br>• Include the approval number and/or a statement indicating approval of this research<br>• Indicate the form of consent obtained (written/oral) or the reason that consent was not obtained (e.g. the data were analyzed anonymously)<br><br>**Animal Research (involving vertebrate animals, embryos or tissues)**<br>• Provide the name of the Institutional Animal Care and Use Committee (IACUC) or other relevant ethics board that reviewed the study protocol, and indicate whether they approved this research or granted a formal waiver of ethical approval<br>• Include an approval number if one was obtained<br>• If the study involved *non-human primates*, add *additional details* about animal welfare and steps taken to ameliorate suffering<br>• If anesthesia, euthanasia, or any kind of animal sacrifice is part of the study, include briefly which substances and/or methods were applied<br><br>**Field Research**<br><br>Include the following details if this study involves the collection of plant, animal, or other materials from a natural setting:<br>• Field permit number<br>• Name of the institution or relevant body that granted permission	
**Data Availability**<br><br>Authors are required to make the data underlying their research findings fully available, without restriction.<br><br>If the manuscript reports pilot or preliminary results, the data underlying those results must be made available at the time of publication. A Data Availability	N/A - No results are reported

Statement describing where the data can be found is required at submission. Your answers to this question constitute the Data Availability Statement and will be published in the article, if accepted. When legal or ethical requirements prohibit public data sharing, authors must indicate how researchers may obtain access to the data. See the PLOS Data Policy and FAQ for detailed information.<br><br>**Important:** Stating 'data available on request from the author' is not sufficient. If your data are only available upon request, select 'No' for the first question and explain your situation in the text box.<br><br>For protocols without pilot or preliminary data, authors are strongly encouraged to state how they plan to share research data from their study when it is completed or published.<br><br>If your study includes pilot data, will they be made fully available without restriction?	
**Describe where the data may be found in full sentences. If you are copying our sample text, replace any instances of XXX with the appropriate details.**<br><br>• If **no pilot data are reported** in the manuscript, please enter the following: *No datasets were generated or analysed during the current study. All relevant data from this study will be made available upon study completion.*<br>• If the manuscript reports pilot data **held in a public repository**, include URLs, accession numbers or DOIs. If this information will only be available after acceptance, indicate this by ticking the box below. For example: *All XXX files are available from the XXX database (accession number(s) XXX, XXX.).*<br>• If the manuscript reports pilot data contained **within the manuscript and/or**	Not applicable

**Supporting Information files**, enter the following:<br>*All relevant data are within the manuscript and its Supporting Information files.*<br>• If neither of these applies but you are able to provide **details of access elsewhere**, with or without limitations, please do so. For example:<br><br>*Data cannot be shared publicly because of [XXX]. Data are available from the XXX Institutional Data Access / Ethics Committee (contact via XXX) for researchers who meet the criteria for access to confidential data.*<br><br>*The data underlying the results presented in the study are available from (include the name of the third party and contact information or URL).*<br>• This text is appropriate if the data are owned by a third party and authors do not have permission to share the data.<br><br>For study protocols with data management or sharing plans, authors are encouraged to briefly describe in their Data Availability Statement how data they generate will be made accessible when the study is completed. For example:<br>• Deidentified research data will be made publicly available when the study is completed and published.<br><br>* typeset	
Additional data availability information:	Tick here if the URLs/accession numbers/DOIs will be available only after acceptance of the manuscript for publication so that we can ensure their inclusion before publication.

Powered by Editorial Manager® and ProduXion Manager® from Aries Systems Corporation

Dear Editor in Chief

Attached please find an electronic file of a manuscript entitled "***Randomized controlled trial of the effectiveness of olive and black seed oil combination in pain intensity and episiotomy wound healing in primiparous women: study protocol***" That we are submitting for review and consideration for publication in your valuable ***Journal.***

I wish to have the manuscript considered as a Study Protocol, and confirm that neither recruitment nor data collection have been completed.

Our study was approved by Ethics Committee of Babol University of Medical Sciences (IR.MUBABOL.REC.1401.145). Also, the study protocol was registered in the Iranian Registry of Clinical Trial (IRCT20180218038783N2). I confirm the study start date/date when participant recruitment started (2022-12-23).

The manuscript has not been and will not be published elsewhere or submitted elsewhere for publication. We hereby transfer, assign, or otherwise convey all copyright ownership, including any and all rights incidental thereto, exclusively to the journal, in the event that such work is published by the journal.

We hope this article be interest to the readers of your journal.

Regards

Fereshteh Behmanesh: PhD of Reproductive Heath. Social Determinants of Health Research Center, Health Research Institute, Babol University of Medical Sciences, Babol, I.R. Iran

Email: f.behmanesh2015@gmail.com

Phone: +98 9112135312

Postal code: 47176-47745

Manuscript

Click here to access/download;Manuscript;olive oil article VANCOUVER-8-8-402.doc

Randomized controlled trial of the effectiveness of olive and black seed oil combination in pain intensity and episiotomy wound healing in primiparous women: study protocol

Romina fili[1], Fereshteh Behmanesh[2*], Maryam Nikpour[3], Zahra Memariani[4], Sana Nazmi[5]

[1] BSc in Midwifery, Faculty of nursing & Midwifery Babol University of Medical Sciences, Babol, I.R. Iran rominafili.mid@gmail.com ORCID:0009-0005-0705-8007

[2*] (correspondence), Assistant Professor of Reproductive Health, Social Determinants of Health Research Center, Health Research Institute, Babol University of Medical Sciences, Babol, I.R. Iran
Email: f.behmanesh2015@gmail.com ORCID: 0000-0001-5837-4265 Tel:09112135312

[3] Assistant Professor of Health Sciences. Non-Communicable Pediatric Disease Research Center, Health Research Institute, Babol University of Medical Sciences, Babol, I.R.Iran Email: maryamnikpour19@yahoo.com ORCID: 0000-0001-6821-0010

[4] Traditional medicine specialist, Traditional Medicine and History of Medical Sciences Research Center, Health Research Institute, Babol University of Medical Sciences, Babol, I.R.Iran
z.memariani@mubabol.ac.ir ORCID:0000-0002-2052-4261

[5] MSc in counseling in Midwifery, Faculty of nursing & Midwifery, Babol University of Medical Sciences, Babol, I.R. Iran sananazmi98@gmail.com ORCID: 0000-0002-7005-2717

Randomized controlled trial of the effectiveness of olive and black seed oil combination in pain intensity and episiotomy wound healing in primiparous women: study protocol

Abstract

Background: Episiotomy is associated with side effects, such as pain and wound infection. Also, Discomfort after episiotomy affects the quality of life of mother and baby. Medicinal herbs are one alternative methods for the treatment of episiotomy wound. This study will investigate the effectiveness of olive and black seed oil combination in pain intensity and episiotomy wound healing in primiparous women.

Methods: This randomized clinical trial will be done with two interventions and one control groups on primiparous women have normal delivery with episiotomy. women in the intervention group (One group, olive oil plus black seed oil and another group olive oil alone) will use a glass containing oils with a dose of 10 drops locally and 3 times a day 24 hours after delivery, for 10 days. The control group will receive only routine care[illegible] Data will be collected through demographic characteristic questionnaire, REEDA scale a[illegible] visual analog scale. In order to determine and compare the effect of pharmacological interventions on the severity of pain and episiotomy wound healing in groups, an ANOVA analysis of variance test with repeated measures will be used by SPSS software version 22.

Discussion: The results of this study will show the effects of olive and black seed oil combination and olive oil alone in pain intensity and episiotomy wound healing in primiparous women. The beneficial effects likely to be found in the trial of these oils may be beneficial for women who have undergone episiotomy.

Trial registration: IRCT20180218038783N2. Registered at 2022-12-23.

Keywords: Olive oil, Black seed oil, Episiotomy, Wound healing, Clinical protocol

Highlights

- Episiotomy is associated with side effects, such as pain and wound infection
- Due to the high costs and more complications of treatment with chemical drugs, it is better to use herbal drugs according to the recommendation of the World Health Organization.
- It seems that plant oils such as olive oil and black seed oil are effective in healing and repairing perineal wounds.

Introduction

Episiotomy is an incision in the area between the vaginal opening and the anus, which is performed to enlarge the vaginal opening in order to facilitate the process of natural childbirth, especially in primiparous women (1-3). Episiotomy is associated with side effects, such as pain (3-5), which studies have shown that approximately 30% of women experience in the first two weeks and 7% up to three months after natural delivery (6). There is a possibility of wound infection due to the lack of direct observation by the mother and the closeness of the wound area to the anus (7-9). Discomfort after episiotomy affects the quality of life of mother and baby (3, 10, 11). Considering that the baby needs the presence and support of the mother for growth and development, especially in the early days of birth, it is important to pay attention and support to mothers who have episiotomy (7, 12).

Since the current policy of Iran is to increase the birth rate and population should be paid more attention to care during and after pregnancy, especially natural childbirth. Because it may cause an unpleasant experience for the mother, which will affect her future pregnancies and lead to not having children and then reducing the growth of the population (6). On the other hand, not performing an episiotomy can lead to more severe tears in the perineal area, which causes problems for the mother. Such as reducing the quality of sex (4, 13) and breastfeeding the baby (2, 14). In order to relieve ~~wound~~ pain and episiotomy repair, different local treatment methods, oral, rectal and sitting bath in hot water are used (12, 15). Due to the Popularization of herbal medicine in recent decades (16) and adverse effects of chemical drugs such as high costs (4) and mild drowsiness to cardiotoxicity (17), , it is better to use herbal drugs according to the recommendation of the World Health Organization (18). As an example of this huge problem, side effects from chemical drugs are reported to be the fourth leading cause of death in the United States. So, it causes the death of 100,000 people a year(17). It seems that plant oils such as olive oil and black seed oil are effective in healing and repairing perineal wounds (18, 19). According to studies, many medicinal plants, especially olive oil, accelerate wound healing and reduce pain and complications caused by episiotomy (14, 20). Olive oil is rich in substances that give it anti-microbial, anti-fungal and anti-inflammatory properties (21, 22). Such as peniphenol, which promotes cell repair, antioxidants that help reduce inflammation, and oleocanthal, which suppresses pain ((12, 20, 23). In addition, various studies have shown the effects of olive oil (24), its unsaturated fatty acids, including oleic acid (25) and its phenolic compounds (26) in wound healing. Olive oil is effective in treating skin diseases, suppressing the growth of viruses and bacteria, and healing wounds (12, 27).

Black seed contains 30 to 40% oil and is rich in a type of antioxidant called thymoquinone (28, 29), phenols, linoleic acid and oleic acid, which various studies have shown its effects in wound healing (30). Also, black seed reduces inflammation and has analgesic, anti-inflammatory, anti-ischemic, antiviral and antifungal effects (31, 32). It can be said that the analgesic, anti-inflammatory, antifungal and antibacterial effects of black seed are related to thymoquinone, which is one of the main constituents of black seed (33). Thymoquinone is a monoterpene diketone with a molecular weight of 164.204 g/mol and is considered a lipophilic compound (34). In fact, most properties of this plant are attributed to thymoquinone (35). Studies have shown that black seed is suitable for wound healing in the human body and can prevent infection (32, 36), and adding black seed oil to olive oil increases its antioxidant properties (37).Considering that not many studies have been conducted in relation to the local effectiveness of the combination of olive oil and black seed oil on pain relief and episiotomy wound healing, and also human clinical studies on black seed oil are limited, the present study aims to determine the effectiveness of olive and black seed oil combination in pain intensity and episiotomy wound healing in primiparous women with a parallel design .

Materials and Methods

Our objectives in this protocol are (1) Comparison of the change in the average intensity of episiotomy pain in three groups receiving the combination of olive oil and black seed, olive oil alone and the routine care group (2) Comparison of the average change of episiotomy wound healing in three groups ~~receiving the combination of olive oil and black seed oil and the routine care group~~ (3) Comparison of side effects caused by drug use (burning and itching, episiotomy wound opening and need for repair) in three groups

This parallel-group, non-inferiority study randomized clinical trial will be performed on postpartum women with episiotomy in Babol University of Medical Sciences hospitals. In this study, the design according to the interpretation standards of the report of trials has been followed (Figure 1). Our study was approved by Ethics Committee of Babol University of Medical Sciences (IR.MUBABOL.REC.1401.145). Also, the study protocol was registered in the Iranian Registry of Clinical Trial (IRCT20180218038783N2).

Women who are interested in participating in the study are invited to participate in this study. After explaining the objectives of the research to the women and obtaining the informed consent of the qualified, the researcher will proceed with convenient sampling. Then, according to the study [illegible]clusion criteria, the samples will be randomly assigned to two intervention groups and one routine care group.

Inclusion criteria ~~will be~~: primiparous women, normal delivery with episiotomy, having literacy and BMI < 30. Exclusion criteria ~~will be~~: The personal unwillingness of the participants to continue the study, Women who do not use oils regularly,4th degree tear, history of diagnosed skin dermatitis, history of allergy and eczema diagnosed to plants, history of gestational diabetes and obvious

[illegible]e researcher will select the samples according to the inclusion criteria and assign them randomly (using the block randomized method) into two groups of intervention and routine care.

Eligible people are placed in one of three groups A: 36 women, B: 36 women, and C: 36 women, using permuted block randomization and open label. The size of the blocks is 6 and in each block each intervention group will be repeated twice. 18 blocks of 6 will be produced to create a sequence of size 108. .he table will be managed by a researcher who is not present in the sampling, and after specifying the sample based on the inclusion criteria by the person who is responsible for the sampling, the intervention code is received from that researcher.

In order to hide the list of random allocation, a special code will be assigned to each of the intervention groups, which only the supervisor will be aware of. The supervisor who is responsible for code generation does not know the type of codes assigned to the three intervention groups. Also, the second moderator, who is a student and the person who performs the statistical analysis, will not know about the codes assigned to the drugs.

This research will be carried out after obtaining permission from the Ethics Committee of Babol University of Medical Sciences and registering in the Iranian Clinical Trial Registration Center (IRCT) and obtaining the necessary permits for sampling. After referring to the midwifery ward of the hospitals covered by Babol University of Medical Sciences, the researcher invites women who meet the inclusion criteria to participate in the study. The researcher will first explain the purpose of the study and the type of intervention for women. Then, he will assure them about the confidentiality of information, the researcher's accountability for causing any side effects in the treatment process and no change in the process of therapeutic interventions in case of not accepting participation in the study. Participants are then asked to sign a written consent form. In addition, participants are assured that they can leave the study at any stage. Then the researcher (student) informs the supervisor of the individual's entry into the study, and the supervisor tells the researcher the relevant intervention code. The supervisor, researcher and statistical analyst will be blinded to perform proper management.

This clinical trial will be done with two interventions and one control groups. three blind strains, 108 randomized samples with blocks of four and open label in three groups. Eligible people will be placed in one of three groups A: 36 women, B: 36 women, and C: 36 women, using permuted block randomization and open label. The size of the blocks is 6 and in each block each intervention group will be repeated twice. 18 blocks of 6 will be produced to create a sequence of size 108. women in the intervention group (One group, olive oil plus black seed oil and another group olive oil alone) will use a glass containing oils with a dose of 10 drops locally and 3 times a day 24 hours after delivery, for 10 days. The control group will receive only routine care.

All mothers will receive painkillers as needed. The type of pain reliever as well as its number per day is evaluated in three groups by a checklist that is completed by the mother within ten days. In this research, three instruments will be used:

1) Demographic characteristics questionnaire

   It consists of 22 questions that include information such as age, education level, place of residence, employment status, height, weight, number of pregnancy and abortion, duration of hospitalization in labor, receiving antibiotics during labor and after delivery,

hospitalization of the baby in the intensive care unit. In fact, the questionnaire was designed based on the research objectives and recognition of confounding variables.

2) visual analog scale that will be used to measure episiotomy pain intensity.
   Visual analog scale or McGill pain ruler is an eye scale that is used to measure pain. This ruler is ten centimeters. Its number zero means no pain and its number 10 means the worst possible pain. Between these two ends, it is divided into three levels of mild pain (1-3), moderate pain (4-7) and severe pain (8-10) (38). Grading will be record by self-report method of pregnant women before and after interventions.
3) REEDA scale
   It is a standard scale for evaluating episiotomy wound healing and includes the variables of swelling, bruising, discharge, redness and the distance between the two edges of the episiotomy wound. Each of these variables is scored from 0 to 3. The scores of each variable will be record separately after observation and examination by the researcher, before and after the examination. The overall score of the questionnaire is from 0 to 15. A higher score indicates less wound healing and a lower score indicates better wound healing. The validity and reliability of this international scale has been recorded (39).

The primary outcome is intensity of pain in the episiotomy area and healing of episiotomy wound which pain intensity will be measured by visual pain scale and episiotomy wound repair by REEDA scale. Burning and itching and episiotomy opening and the need for painkillers which will be evaluated by a checklist. The side effects reported by each sample and the total number of side effects for each group will be reviewed.

To prepare the product, cold-pressed black seed oil is used, as well as standard extra-virgin olive oil (according to the national standard). Extra virgin standard olive oil is prepared by a pharmaceutical grade company (Pishgaman Chemi). Cold-pressed black seed oil is obtained from pharmaceutical company (Barij Essance). Both oils are mixed and packaged in equal proportions (40). Both products have approved specifications in terms of microbial and pollution control standards, chemical and physical control. The process of mixing and packaging of two oils is done under hygienic conditions in the traditional pharmacy laboratory of the Faculty of Traditional Medicine.

Intervention group 1: recipient of black seed oil and olive: All the subjects of the study will be taught how to follow the health tips after childbirth, and the samples of the intervention group will be taught how to use the container containing the oils. In such a way that they use the container containing black seed and olive oil with a dose of 10 drops locally and three times a day 24 hours after giving birth, for 10 days. The researcher also examines the severity of perineal pain and healing of episiotomy wound in the first 24 hours after delivery and ten days after that. Control group receive routine cares.

The sample size was calculated at the confidence level of 95%, the power of the test was 80% and according to the information of the article by Barati et al. (18) with the values of $\sigma 1=1.5$, $\mu 1=5$, $\sigma 2=2.2$, and $\mu 2=6.4$, and with the assumption of spillage during execution., the number of 36 samples in each group is estimated. The analysis method in this study is intention-to-treat analysis in order to maintain the random allocation process. After collecting the data in the first

stage, the basic variables of the patients in the intervention and control groups will be compared to ensure the desirability of the random allocation process. SPSS version 22 software and ANOVA, chi-square, repeated measurements and multilevel mixed-effects linear regression will be used to analyze the primary results. The results will be calculated and reported as adjusted mean difference with 95% confidence limits. The variables included in the model include all the variables affecting the outcome. A significance level of 0.05 will be considered in all tests.

Researchers increase contact with individuals to optimize completeness of data collection. For this purpose, we will strengthen communication with the samples through social media and phone to remind them of their medications. One of the researchers (RF) will be responsible for data collection and appoint another person (SN) to enter information into SPSS software. A data manager (MN) examines all data without knowing the treatment allocation. These cases are done under the supervision of FB.

Ethical considerations

The study is approved by the Ethics Committee of Babol University of Medical Sciences (IR.MUBABOL.REC.1401.145). All methods will perform in accordance with the relevant guidelines and regulations (Declaration of Helsinki) and written informed consent obtained from participants.

Discussion

Olive oil is rich in substances that give it anti-microbial, anti-fungal and anti-inflammatory properties (22, 41). Such as peniphenol, which promotes cell repair, antioxidants that help reduce inflammation, and oleocanthal, which suppresses pain (12, 20, 23). Black seed also contains 30 to 40% oil and is rich in a type of antioxidant called thymoquinone (28, 29), phenols, linoleic acid and oleic acid, which various studies have shown to be effective in wound healing (30). Studies show that black seed is suitable for wound healing in the human body and can prevent the occurrence of infection (32, 36), and adding black seed oil to olive oil increases its antioxidant properties (37).

The effect of black seed (18) and olive oil (6, 12) alone on episiotomy has been evaluated in different studies. But based on the research done by the research team, this study is the first study that will examine the effect of the combination of these two oils on episiotomy. There is little clinical research on whether topical use of a combination of these two oils can be effective on episiotomy repair and perineal pain severity or not. If the results of the present study show that the combination of these two substances can be effective on the severity of episiotomy pain and repairing, it can be suggested as a cost-effective treatment.

Conclusion

The results of this study will show the effects of olive and black seed oil combination and olive oil alone in pain intensity and episiotomy wound healing in primiparous women. The beneficial effects likely to be found in the trial of these oils may be beneficial for women who have undergone episiotomy.

Declarations

Ethics approval and consent to participate

The study is approved by the Ethics Committee of Babol University of Medical Sciences (IR.MUBABOL.REC.1401.145). All methods will perform in accordance with the relevant guidelines and regulations (Declaration of Helsinki) and writhen informed consent obtained from participants. The results of the trial will be communicated to participants, health care professionals, the public and other relevant groups, researchers and sponsors through publication of results and reporting in results databases.

Consent for publication

Not applicable

Availability of data and materials

Not applicable

Competing interests

The authors have declared that no competing interests exist.

Funding

This study was financially supported by Babol University of Medical Sciences. Award Number: 724134467.

Authors' contributions

F.B. & R.F. & Z.M. Conceptualization, Methodology, Software, M.N. Validity tests, Data curation, Writing- Original draft preparation. R.F. & S.N. Visualization, Investigation. F.B. Supervision. R.F. Software Validation. F.B. & R.F. Writing- Reviewing and Editing.

Acknowledgements

The authors would like to thank the Deputy of Research and Technology of Babol University of Medical Sciences for supporting the project.

References

1. Tara F, Golmakani N, Motlagh ER, Assili J, Shakeri M. P284 The effects of turmeric (Curcuma longa L) ointment on healing of episiotomy site in primiparous women. International Journal of Gynecology & Obstetrics. 2009;107:S493-S4.
2. Vakilian K, Atarha M, Bekhradi R, Chaman R. Healing advantages of lavender essential oil during episiotomy recovery: a clinical trial. Complementary therapies in clinical practice. 2011;17(1):50-3.
3. Moradi M, Niazi A, Mazloomi E, Mousavi SF, Lopez V. Effect of lavender on episiotomy wound healing and pain relief: a systematic review. Evidence Based Care Journal. 2020;10(1):61-9.
4. Sabzaligol M, Safari N, Baghcjeghi N, Latifi M, Bekhradi R, Taghizadeh M, et al. The effect of Aloevera gel on prineal pain & wound healing after episiotomy. Complementary Medicine Journal. 2014;4(2):766-75.
5. Shayan A, Sourinezhad H, Barzegar F, Ahmadinia H, Masoumi Z, Moradkhani S, et al. The effect of olive oil and honey combination on episiotomy wound healing and pain relief: A randomized clinical trial. Current Women's Health Reviews. 2020;16(2):145-51.
6. Hables RM. Effect of Olive Oil, Lavender Oil and Placebo on Pain Intensity and Healing of Episiotomy in Women. Tanta Scientific Nursing Journal. 2021;20(1):47-63.
7. Mardani F, Hadizadeh-Talasaz F, Bahri N. Effect of medicinal plants on episiotomy wound healing in Iran: a systematic review study. The Iranian Journal of Obstetrics, Gynecology and Infertility. 2020;23(5):79-94.
8. Nikpour M, Delavar MA, Khafri S, Ghanbarpour A, Moghadamnia AA, Esmaeilzadeh S, et al. The use of honey and curcumin for episiotomy pain relief and wound healing: A three-group double-blind randomized clinical trial. Nursing and Midwifery Studies. 2019;8(2):64.
9. Torkashvand S, Jafarzadeh-Kenarsari F, Donyaei-Mobarrez Y, Chaboki BG. Effectiveness of Olea Herbal Ointment on Episiotomy Wound Healing Among Primiparous Women: A Randomized Clinical Trial. Jundishapur Journal of Natural Pharmaceutical Products. 2021;16(3).
10. Sheikhan F, Jahdi F, Khoei EM, Shamsalizadeh N, Sheikhan M, Haghani H. Episiotomy pain relief: Use of Lavender oil essence in primiparous Iranian women. Complementary therapies in clinical practice. 2012;18(1):66-70.
11. Golozar S, Namjouian F, Latifi SM, Mirahi A. Evaluating the effect of oral bromelain (pineapple) on episiotomy wound healing in primiparus women. KAUMS Journal (FEYZ). 2011;15(2):84-90.
12. Behmanesh F, Aghamohammadi A, Zeinalzadeh M, Khafri S. Effects of olive oil sitz bath on improvement of perineal injury after delivery. Koomesh. 2013;14(3).
13. Hartmann K, Viswanathan M, Palmieri R, Gartlehner G, Thorp J, Lohr KN. Outcomes of routine episiotomy: a systematic review. Jama. 2005;293(17):2141-8.
14. Kaviani M, Sepasi S, Azima S, Emamghoreishi M, Asadi N, Haghpanah S. The effects of olive leaf extract ointment on pain intensity and early maternal complications in primiparous women. Int J Pharm Pharm Sci. 2017;9(7):31-4.
15. Mohamed H, El-Nagger NS. Effect of self perineal care instructions on episiotomy pain and wound healing of postpartum women. J Am Sci. 2012;8(6):640-50.
16. Ahad B, Shahri W, Rasool H, Reshi Z, Rasool S, Hussain T. Medicinal plants and herbal drugs: An overview. Medicinal and Aromatic Plants: Healthcare and Industrial Applications. 2021:1-40.
17. Huang L-C, Wu X, Chen JY. Predicting adverse side effects of drugs. BMC genomics. 2011;12(5):1-10.
18. Barati N, Kordi M. The effect of black seed cream on episiotomy pain in primiparous women: a clinical trial study. The third international conference on new findings in obstetrics, gynecology, childbirth and infertility2020.

19. Amani R, Kariman N, Mojab F, Alavi H, Majidi S. Comparison of the effects of cold compress with gel packs and topical olive oil on episiotomy wound healing. Journal of Babol University of Medical Sciences. 2015;17(11):7-12.
20. Abooei Mehrizi M, Eidi A, Mortazavi P. Study of effect of olive oil on re-epithelialization of epithelial tissue in excision wound healing model in rats. Journal of Comparative Pathobiology. 2016;13(2):1875-84.
21. Preedy VR, Watson RR. Olives and olive oil in health and disease prevention: Academic press; 2020.
22. Donato-Trancoso A, Monte-Alto-Costa A, Romana-Souza B. Olive oil-induced reduction of oxidative damage and inflammation promotes wound healing of pressure ulcers in mice. Journal of dermatological science. 2016;83(1):60-9.
23. Raisi A, Farjanikish G, Salahi P. Comparing Healing Effects of olive leaf extract ointment and dermaheal ointment on cutaneous wound in diabetic rats. Journal of Mazandaran University of Medical Sciences. 2019;29(173):11-21.
24. Taheri M, Amiri-Farahani L. Anti-Inflammatory and Restorative Effects of Olives in Topical Application. Dermatology Research and Practice. 2021;2021.
25. Sales-Campos H, Reis de Souza P, Crema Peghini B, Santana da Silva J, Ribeiro Cardoso C. An overview of the modulatory effects of oleic acid in health and disease. Mini reviews in medicinal chemistry. 2013;13(2):201-10.
26. Carrara M, Kelly MT, Roso F, Larroque M, Margout D. Potential of olive oil mill wastewater as a source of polyphenols for the treatment of skin disorders: a review. Journal of Agricultural and Food Chemistry. 2021;69(26):7268-84.
27. Bayir Y, Un H, Ugan RA, Akpinar E, Cadirci E, Calik I, et al. The effects of Beeswax, Olive oil and Butter impregnated bandage on burn wound healing. Burns. 2019;45(6):1410-7.
28. Randhawa MA, Alghamdi MS. Anticancer activity of Nigella sativa (black seed)—a review. The American journal of Chinese medicine. 2011;39(06):1075-91.
29. Vedaei S, Aarabi A. An investigation on the effect of cold press and soxhlet methods of extracting Iranian black seed oil on physicochemical and antioxidant properties. Journal of Food Processing and Preservation. 2021;13(1):89-101.
30. Sallehuddin N, Nordin A, Bt Hj Idrus R, Fauzi MB. Nigella sativa and its active compound, thymoquinone, accelerate wound healing in an in vivo animal model: A comprehensive review. International journal of environmental research and public health. 2020;17(11):4160.
31. Noorbar E, Mirazi N. Study of Nigella sativa L. seed's hydroethanolic extract on skin wound healing in male diabetic rats. Armaghane Danesh. 2017;22(4):419-30.
32. Gumus ZP, Guler E, Demir B, Barlas FB, Yavuz M, Colpankan D, et al. Herbal infusions of black seed and wheat germ oil: Their chemical profiles, in vitro bio-investigations and effective formulations as phyto-nanoemulsions. Colloids and Surfaces B: Biointerfaces. 2015;133:73-80.
33. Shaterzadeh-Yazdi H, Noorbakhsh M-F, Samarghandian S, Farkhondeh T. An overview on renoprotective effects of thymoquinone. Kidney Diseases. 2018;4(2):74-82.
34. Pottoo FH, Ibrahim AM, Alammar A, Alsinan R, Aleid M, Alshehhi A, et al. Thymoquinone: Review of its potential in the treatment of neurological diseases. Pharmaceuticals. 2022;15(4):408.
35. Tavakkoli A, Mahdian V, Razavi BM, Hosseinzadeh H. Review on clinical trials of black seed (Nigella sativa) and its active constituent, thymoquinone. Journal of pharmacopuncture. 2017;20(3):179.
36. Zaid MHM, Hanafi MF, Haris MS. A review of black seed extract as an agent in the wound healing process. Journal of Pharmacy. 2021;1(2):87-96.
37. Shaheen M, Dolganova N, Shinkar EV, Sukhenko LT, Astafieva OV. STUDY OF BIOTECHNOLOGY RAISE ANTIOXIDANT PROPERTIES OF OLIVE OIL AND BLACK SEED OIL. Carpathian Journal of Food Science & Technology. 2020;12(2).
38. Hekmatzadeh S, Mirmolaei S, Hoseini N. The effect of boiled dill (Anethum graveolens) seeds on the long active phase and labor pain intensity. Armaghane danesh. 2012;17(1):50-9.
39. Hill PD. Psychometric properties of the REEDA. Journal of nurse-midwifery. 1990;35(3):162-5.

40. Yaman I, Durmus A, Ceribasi S, Yaman M. Effects of Nigella sativa and silver sulfadiazine on burn wound healing in rats. Veterinarni Medicina. 2010;55(12):619-24.
41. Victor R Preedy RW. Olives and Olive Oil in Health and Disease Prevention. 2nd Edition ed2020 December 2. 746 p.

Figure1. The schedule of enrolment, interventions, and assessments

	STUDY PERIOD							
	**Enrolment**	**Allocation**	**Post-allocation**					**Close-out**
**TIMEPOINT****	T_1	*0*	10th *day*					2024 December
**ENROLMENT:**								
**Eligibility screen**	X							
**Informed consent**	X							
**Allocation**	X							
**INTERVENTIONS:**	X							
[Olive Oil]	X	X	↔					
[olive oil plus black seed oil]	X	X	↔					
[Control]	X	X	↔					
**ASSESSMENTS:**								
[intensity of pain in the episiotomy area and healing of episiotomy wound]	X		X					X
[side effects: (burning and itching, episiotomy wound opening and need for repair, The need for painkillers)]	X		X					X
[Demographic variables]		X						X

Click here to access/download
Supporting Information
Olive Proposal -latin.docx

Click here to access/download
Supporting Information
Olive & black seed proposal 11-7-401.docx

SPIRIT

Click here to access/download
Supporting Information
Olive -SPIRIT-Checklist3-8-402.doc

Ethic letter

Click here to access/download
Other
ethic olive-latin.pdf
