# Supplementary material for: Randomized controlled trial of the effectiveness of olive and black seed oil combination on pain intensity and episiotomy wound healing in primiparous women: A study protocol
Source: PLoS One. 2024 May 15;19(5):e0302161. doi: 10.1371/journal.pone.0302161 (PMC11095949; doi:10.1371/journal.pone.0302161)
Supplement: Attachment — Submitted filename: Response to reviewers.docx [file pone.0302161.s004.docx]

**Dear editor**

**PLOS ONE**

Thank you and the honorable Reviewer (s) for your valuable comments for the manuscript entitled: **Randomized controlled trial of the effectiveness of olive and black seed oil combination on pain intensity and episiotomy wound healing in primiparous women: study protocol (PONE-D-23-34640).** These comments are very useful in the scientific improvement of the manuscript. Responses to the following comments are given:

1. Improve the general outlook of the protocol by restructuring sentences, make your statement clear and logical to enable your readers understand your work. Also, attend to incomplete sentences to use of words.

Regards, corrected.

2. Make protocol succinct and avoid repetitions.

These items have been corrected and marked in the text with track changes.

3. The "Material and methods" section can be structured to reflect the following:

Study design

Eligibility criteria

Study sample and sampling

Data collection and management

Data analysis

Report

Sincerely, corrected.

4. Please include the following items when submitting your revised manuscript:

A rebuttal letter that responds to each point raised by the academic editor and reviewer(s). You should upload this letter as a separate file labeled 'Response to Reviewers'.

Done

A marked-up copy of your manuscript that highlights changes made to the original version. You should upload this as a separate file labeled 'Revised Manuscript with Track Changes'.

Done

An unmarked version of your revised paper without tracked changes. You should upload this as a separate file labeled 'Manuscript'.

Done

If you would like to make changes to your financial disclosure, please include your updated statement in your cover letter.

Corrected.

Journal Requirements:

When submitting your revision, we need you to address these additional requirements.

1. Please ensure that your manuscript meets PLOS ONE's style requirements, including those for file naming. The PLOS ONE style templates can be found at

https://journals.plos.org/plosone/s/file?id=wjVg/PLOSOne_formatting_sample_main_body.pdf and

<https://journals.plos.org/plosone/s/file?id=ba62/PLOSOne_formatting_sample_title_authors_affiliations.pdf>

This manuscript meets PLOS ONE's style requirements, including those for file naming.

2. Thank you for stating in your Funding Statement:

"This study was financially supported by Babol University of Medical Sciences. Award Number: 724134467. F.B will receive the fund.

URL: https://www.mubabol.ac.ir/

This funder contributed to the approval of the study."

Please provide an amended statement that declares *all* the funding or sources of support (whether external or internal to your organization) received during this study, as detailed online in our guide for authors at http://journals.plos.org/plosone/s/submit-now. Please also include the statement “There was no additional external funding received for this study.” in your updated Funding Statement.

Please include your amended Funding Statement within your cover letter. We will change the online submission form on your behalf.

Corrected

3. Thank you for stating the following in the Acknowledgments Section of your manuscript:

"The authors would like to thank the Deputy of Research and Technology of Babol University of Medical Sciences for supporting the project."

We note that you have provided funding information that is not currently declared in your Funding Statement. However, funding information should not appear in the Acknowledgments section or other areas of your manuscript. We will only publish funding information present in the Funding Statement section of the online submission form.

Please remove any funding-related text from the manuscript and let us know how you would like to update your Funding Statement. Currently, your Funding Statement reads as follows:

"This study was financially supported by Babol University of Medical Sciences. Award Number: 724134467. F.B will receive the fund.

URL: https://www.mubabol.ac.ir/

This funder contributed to the approval of the study."

The Acknowledgments Section corrected: The authors would like to thank the Deputy of Research and Technology of Babol University of Medical Sciences and mothers who will participate in this study.

Please include your amended statements within your cover letter; we will change the online submission form on your behalf.

The amended statements was included in the cover letter.

4. We note that you have stated that you will provide repository information for your data at acceptance. Should your manuscript be accepted for publication, we will hold it until you provide the relevant accession numbers or DOIs necessary to access your data. If you wish to make changes to your Data Availability statement, please describe these changes in your cover letter and we will update your Data Availability statement to reflect the information you provide.

This is a protocol study and data availability are not applicable. This sentence is included in the manuscript and cover letter.

5. Please note that in order to use the direct billing option the corresponding author must be affiliated with the chosen institute. Please either amend your manuscript to change the affiliation or corresponding author, or email us at plosone@plos.org with a request to remove this option.

I emailed to plosone@plos.org with a request to remove the direct billing option.

6. Your ethics statement should only appear in the Methods section of your manuscript. If your ethics statement is written in any section besides the Methods, please delete it from any other section.

Corrected. It deleted from declarations.

7. We note that the original protocol that you have uploaded as a Supporting Information file contains an institutional logo. As this logo is likely copyrighted, we ask that you please remove it from this file and upload an updated version upon resubmission.

I removed it from the original protocol and uploaded an updated version upon resubmission.

8. Please include captions for your Supporting Information files at the end of your manuscript, and update any in-text citations to match accordingly. Please see our Supporting Information guidelines for more information: <http://journals.plos.org/plosone/s/supporting-information>.

Corrected based on Supporting Information guidelines.
