# Supplementary material for: Randomized controlled trial of the effectiveness of olive and black seed oil combination on pain intensity and episiotomy wound healing in primiparous women: A study protocol
Source: PLoS One. 2024 May 15;19(5):e0302161. doi: 10.1371/journal.pone.0302161 (PMC11095949; doi:10.1371/journal.pone.0302161)
Supplement: Attachment — Submitted filename: Response to Reviewers 25-10-402.docx [file pone.0302161.s005.docx]

**Dear Editor in Chief**

**PONE-ONE**

Thank you for your thorough review and consideration of our manuscript “**Randomized controlled trial of the effectiveness of olive and black seed oil combination on pain intensity and episiotomy wound healing in primiparous women: Study protocol**" (PONE-D-23-34640R1) that was submitted to the PONE-ONE.

We believe that the comments and suggestions that were recommended by the reviewers have informed a much improved and more fully developed paper that will offer an important contribution to the field. We have highlighted the changes that were undertaken in response to your comments in the revised manuscript. Responses to each of your comments are below.

Thanks for your kind attention to the manuscript.

Sincerely, Corresponding author

Dr Fereshteh Behmanesh

Reproductive Health

| **Reviewers** | **Response** |
| --- | --- |
| ***Reviewer 1*** | |
| The manuscript has valuable results but needs essential edition as below:  1- English writing is poor. There are a lot of spelling and grammar errors and editing by a native speaker is necessary. | Thank you very much for the opinion of the respected reviewer, according to the suggestion of dear reviewer, this manuscript will be edited by a native and the certificate will be sent for Journal. |
| 2- In the introduction:  • The mention that not performing an episiotomy leads to severe tearing is not true in all mothers and depends on the case. | Corrected, p 3, par 1 |
| In reference number 6, the sentence is incomplete. | Corrected, p 3, par 2 |
| Start sentence with “such as” is unusual. | Corrected, p 4, line 1 & p10, par 3 |
| In the methods section:  • First, the type of study and then the work method should come. | First, the type of study and then the work method writhed. P 5, par 2 |
| Objectives should be removed from the beginning of the methods section. | Removed and the third objective added in the end of introduction. P 5, par 1 |
| • In reliability and validity of Reeda tool, please wright the number of reliability and validity. | Corrected. P 8 & 9 |
| In inclusion criteria: the sentence is incomplete. | Corrected. P6, par 2 |
| Discussion:  • Discussion needs to an essential revision. | In the protocol study, considering that the findings are not clear, the discussion cannot be written in detail. However, it was corrected according to the comment of the respected referee. |
| According to the results, conclusion need revision. | Corrected. P11, par 2 |
| References:  • Some references should be changed basis on guideline format.  • Revise the reference list according to Paper Submission Guide: Abstract the name of journals.  • More references should be covering the last 5 years. | Corrected based on guideline format. |
| ***Reviewer 2*** | |
| First of all this study has some grammatical and typo errors and should be revised by an expert person in English literature. | Thank you very much for the opinion of the respected reviewer, according to the suggestion of dear reviewer, this manuscript will be edited by a native and the certificate will be sent for Journal. |
| Abstract:  1. Method: While one group will receive olive oil plus black seed and one group will receive olive oil alone, why the third group do not receive placebo, and they will receive routine care? | This is a very good question. By the way, the research team had also thought about this issue. But since the use of any oil may have a positive or negative effect on wound healing, for this reason placebo was not used in the control group. |
| Introduction 1. The second point of "highlight" should be revised, as the main reason for using herbs is their safety and not the high costs of pharmaceutical treatment. | With respect to the reviewer’s comment, corrected. P 3 |
| 2. Authors stated that if not using the episiotomy, there is a perineal tears that may cause problem for mother. Non-indicated episiotomy causes many problems for mother such as sexual dysfunction and pain. Please mention the rate of episiotomy especially non-indicated in Iran. | With respect to the reviewer's comment, unfortunately, the incidence of non-indicated episiotomy in Iran was not found in our search. Perhaps because if an episiotomy is non-indicated, the doctor or midwife will not report it due to legal claims.  The following sentence was used instead: In Iran, episiotomy is still common and its prevalence is reported in more than 41% of primiparous women. |
| 3. Please be careful and exact when you are talking about side effects of medications. After delivery, women just receive an analgesic for reducing their pain and analgesics are not expensive and also do not have many side effects. | Thanks to the reviewer, this sentence was removed. Of course, in Iran, in addition to painkillers, most doctors routinely prescribe antibiotics after delivery with episiotomy. |
| 4. Please use the results of those studies that used olive oil for reducing pain intensity after episiotomy in the introduction. | Added. P4, par 2 |
| Methods  1. Women who do not use the oil regularly is not exclusion criteria, but they should consider drop-out. | While thanking the respected reviewer, since our analysis method is ITT, the women should consider drop-out based on reviewer comment. This exclusion criteria were written incorrectly in the manuscript and has now been removed. P 6 |
| 2. Please move the objectives of the study to the end of introduction. | Removed and the third objective added in the end of introduction. P 5, par 1 |
| 3. Some of exclusion criteria are drop-out that happen during study such as "women who do not use olive oil regularly" | While thanking the respected reviewer, since our analysis method is ITT, the women should consider drop-out based on reviewer comment. This exclusion criteria were written incorrectly in the manuscript and has now been removed. P 6 |
| 4. Authors stated that they excluded fourth degree tears. Is that mean you will recruit second and third degree tears? | Yes, that is right. We will recruit second- and third-degree tears. |
| 5. Please provide more details about intervention, e.g. what should participants do before applying the oil on the perineum, any washing? | Provided. The samples were asked to wash and dry their perineum before using oils. Then wear gloves and slowly pour 10 drops of oil on the perineum and massage with hands. P 8, par 1 |
| 6. Please write how much oil will be given to each participant? | Considering that each cc contains 15 drops and mothers should use oils three times a day, approximately 30 cc of each type of oil was given to mothers for 10 days. P 8, par 1 |
| 7. The midwife or a gynecologist that perform episiotomy and type of repair are important factors that did not mention by authors in this study. | The type of repair will be evaluated by questionnaire but the midwife or a gynecologist that perform episiotomy will not be checked. However, in the author's previous study, this issue was investigated and had no effect on the results of the study. |
| 8. Authors should advise participants to take the similar pain killers | The type of painkiller and its number per day in three groups is evaluated by a checklist that is completed by the mother within ten days and then evaluated and considered as auxiliary variable in the final analysis. |
| 9. A significance level of 0.05 will be considered in all tests. It should be <0.05. | corrected |
| **Reviewer 3** | |
| This is an interesting study looking at the effect of olive oil and black seed on improving on pain intensity and episiotomy wound healing in primiparous women. Some comments where the details reported could be clarified further.  1. As this a non-inferiority study, the sample size is missing some fundamental information, i.e what was defined as the clinical minimal difference that would constitute non-inferior. Type 1 error information should be included in sample size calculation. In addition as this is a 3 arms trial, is the non-inferiority hypothesis in relation to control vs EACH intervention, make this comparison explicit and define this. | Thank you very much for the comments of the respected reviewer  According to the suggestion of dear reviewer. Sample size corrected |
| 2. For the randomisation process, please indicate the allocation ratio. No need to state the "The size of the blocks is 6, and 18 blocks of 6 are produced to create a sequence of size 108". You could just state, variable block size. And perharps include this here " (One group, olive oil plus black seed oil and another group of olive oil alone) and control group is routine care" | According to the suggestion of dear reviewer, corrected |
| 3. Unsure what this sentence means "The supervisor, researcher and statistical analyst will be blinded to perform proper management." Do you mean blinded analysis? | As in this sentence: the statistical analyst does not know about the study groups and it will be determined after the statistical analysis (only the pharmacist knows) |
| 4. Define primary outcome, i.e pain intensity- how is this measured? How will healing be measured? | The measurement of pain intensity and wound healing is written in detail in the subtitle of "data collection and management" of the method section in paragraphs 5 and 6.  According to the suggestion of dear reviewer, definition of pain intensity and wound healing was added.  The intensity of perineal pain is the pain that mothers feel in their perineal area and it is recorded by self-report method before and after the intervention.  Skin wound healing is an essential physiological process that consists of the cooperation of many cell strains and their products. Attempts to repair the lesion caused by local invasion begin very early in the inflammatory phase. Finally, they lead to repair, which involves the replacement of specialized structures caused by collagen deposition and regeneration, which is related to the process of cell proliferation and posterior differentiation through cells already present in tissue cells. |
| 5. Same comment for Secondary outcomes, how will these be defined? | The primary outcomes are pain intensity in the episiotomy area and healing of episiotomy wound. The secondary outcomes are Burning and itching and episiotomy opening and the need for painkillers which will be evaluated through examination by the researcher and through mother's self-report. |
| 6. Define what you mean by intention to treat analysis, i,e people analysed according to the group there were assigned regardless of taking allocated intervention. | We will use intention-to-treat analysis in this study, because intention-to-treat analysis will provide an unbiased estimate of the efficacy of the intervention at the level of adherence in the study. |
| 7. More information in the analysis section, i.e mention that data will be reported to CONSORT guidelines as this an RCT. | Statistical analysis according to CONSORT guidelines was written. |
| 8. In the analysis section as this an RCT, no need to carry out formal tests on baseline characteristics as this not recommended, since any differences would occur by chance. Also mention that all details regarding analysis will be stated in the statistical analysis plan. | According to the suggestion of dear reviewer, this sentence was removed |
| 9. What is justification of using multi-level modelling? I.e what are the repeated measures of random effect variable? | Multilevel linear regression will be done for adjust confounders variables (demographic, midwifery and delivery). In the section of statistical analysis was added and repeat measurement analysis was removed. |
| 10. How will adherence/compliance be assessed? | Multilevel linear regression will be done for adjust confounders variables (demographic, midwifery and delivery). |

- A marked-up copy of your manuscript that highlights changes made to the original version. You should upload this as a separate file labeled 'Revised Manuscript with Track Changes'.
- An unmarked version of your revised paper without tracked changes. You should upload this as a separate file labeled 'Manuscript'.
