# Supplementary material for: Randomized controlled trial of the effectiveness of olive and black seed oil combination on pain intensity and episiotomy wound healing in primiparous women: A study protocol
Source: PLoS One. 2024 May 15;19(5):e0302161. doi: 10.1371/journal.pone.0302161 (PMC11095949; doi:10.1371/journal.pone.0302161)
Supplement: Attachment — Submitted filename: Response to Reviewers2.docx [file pone.0302161.s006.docx]

**Dear Editor**

**PONE-ONE**

Thank you for your thorough review and consideration of our manuscript “**Randomized controlled trial of the effectiveness of olive and black seed oil combination on pain intensity and episiotomy wound healing in primiparous women: Study protocol**" (PONE-D-23-34640R1) that was submitted to the PONE-ONE.

We believe that the comments and suggestions that were recommended by the reviewers have informed a much improved and more fully developed paper that will offer an important contribution to the field. We have highlighted the changes that were undertaken in response to your comments in the revised manuscript. Responses to each of your comments are below:

Thanks for your kind attention to the manuscript.

Sincerely, Corresponding author

Dr Fereshteh Behmanesh

Reproductive Health

| **Reviewers** | **Response** |
| --- | --- |
| ***Reviewer #3:*** | |
| All comments have been addressed. | Thank you very much for your valuable comments in the previous review. |
| ***Reviewer #4*** |  |
| 1-Report rate of episiotomy in Iran? | Thank you very much for the attention of the honorable reviewer, rate of episiotomy in Iran added to the introduction. (P4, Par 2) |
| 2- In the introduction: Explain setting of the study and how used type of oil therapy people (primiparous) based on culture. | Corrected (P 6) |
| There are many confounding variables which must be controlled. In Iranian hospitals, the most childbirth is performed by gynecological assistants or midwifery students, with many interventions; confounding variables: for example, time and type of Epi repair, the number of cutgut chromic sutures, Length and depth of episiotomy. fetal information: fetal weight, fetus position of the occiput posterior or anterior, labor information: duration of the first and second stage, removal of the placenta by hand and etc. | You are absolutely right. But as you know, not all interfering factors can be controlled in studies. One of the ways to reduce the effect of these interfering factors is random sampling, which will be considered in this study. However, many of these factors are investigated by the research team. |
| Isn’t report the type of random allocation process. | Thanks to the accuracy of the reviewer, it was corrected (P8, Par 3). |
| How will adherence/compliance be assessed? | In this study, we will examine the adherence of the samples to drug use and side effects. As mentioned in the method, the use of medicine, painkillers and complications of the intervention will be recorded by the mothers within ten days by means of a designed checklist. Also, during two to three phone calls, in these ten days, they are emphasized about the use of oils. (p11, par 2) |
| How many times a day were used the oils? | Oils will be used 3 times a day (p9, par 2) |
| Was it used based on individual demand and then will be recorded. | After explaining the objectives of the research, any mother who is willing to participate in the study and meets the inclusion criteria will be included. The number of mothers who don’t like to participate in this study, will be recorded.(p9, par 1) |
| Isn’t report Reliability this research. | Reliability of this research added to the method. To ensure the reliability of this research, the person who examines the episiotomy wound on the first and tenth days will be the same for all mothers.(p 8, par 4) |
| From the ethical point of view, all three groups should have received painkiller, it should be reported which group used less. | Thank you very much for your accuracy, you are absolutely right. This was written in the manuscript. All three groups can use painkillers. Also, people report how many painkillers they used in ten days by self-reporting through a checklist provided to them. This is mentioned in the method. (p 9, par 3) |
| Report of side effect essential oils? | The side effects of essential oils will be assessed and reported. This is mentioned in the method. (p 11, par 2) |
| when Intensity pain and wound healing will be measured? | In the first 24 hours after delivery, and ten days later. This corrected in the method. (p9, the last paragragh) |
| How Intensity pain and wound healing will be measured? in REEDA scale for evaluate items; sterile tapes should be used to mark the amount of bruising and redness and... then measure with a ruler. | Pain intensity will be measured by VAS based on mother’s self-reporting. About REEDA scale, You are right. But because of the limitations of using sterile tapes in our research, we will use visual inspection. |
| Limitation? For example, this primiparous they change our location during first days, Especially the tenth day, from the mother's house to her house and they are not present at the previous address. | On the 10th day, mothers will be gone to the women's clinic of the hospital for examination. (On the 10th day, gynecologists routinely examine mothers' sutures. Therefore, the address of the mother is not important for us. But one of the major limitations of the current study is that some mothers may not return on the 10th day due to the recovery or because of hospitalization of the baby. This restriction was added to the end of the discussion. |
| Reviewer #5: |  |
| Thank you very much for giving me the opportunity to review this manuscript.  I congratulate the authors for the work done.  I don't have comment. | Thank you very much for your valuable comment. |
