# Supplementary material for: Randomized controlled trial of the effectiveness of olive and black seed oil combination on pain intensity and episiotomy wound healing in primiparous women: A study protocol
Source: PLoS One. 2024 May 15;19(5):e0302161. doi: 10.1371/journal.pone.0302161 (PMC11095949; doi:10.1371/journal.pone.0302161)
Supplement: Attachment — Submitted filename: Response to Reviewers 4.docx [file pone.0302161.s007.docx]

**Dear Editor**

**PONE-ONE**

Thank you for your thorough review and consideration of our manuscript “**Randomized controlled trial of the effectiveness of olive and black seed oil combination on pain intensity and episiotomy wound healing in primiparous women: Study protocol**" (PONE-D-23-34640R1) that was submitted to the PONE-ONE. Thanks for your kind attention to the manuscript.

I review the reference list to ensure that it is complete and correct.

Sincerely, Corresponding author

Dr Fereshteh Behmanesh

PhD in Reproductive Health

| **Reviewers** | **Response** |
| --- | --- |
| ***Reviewer #4*** | |
| Dear authors Thank you for your revises. Please correct these two comments as well:  • you record: the number of the painkiller used during 10 days. | Many thanks for your valuable comments.  Corrected. P 9. Par 3 |
| • you add in the limitations that to assess wound healing, sterile tape was not used and visual inspection was used. | With many respects to you, this sentence added to the limitation.  P 14, par 3 |
